# A long-lasting wireless stimulator for small mammals

**DOI:** 10.3389/fneng.2013.00008

**Published:** 2013-10-11

**Authors:** Ian D. Hentall

**Affiliations:** Department of Neurological Surgery and The Miami Project to Cure Paralysis, Miller School of Medicine, University of MiamiMiami, FL, USA

**Keywords:** deep brain stimulation (DBS), rodents, chronic effects, wireless implant, brainstem

## Abstract

The chronic effects of electrical stimulation in unrestrained awake rodents are best studied with a wireless neural stimulator that can operate unsupervised for several weeks or more. A robust, inexpensive, easily built, cranially implantable stimulator was developed to explore the restorative effects of brainstem stimulation after neurotrauma. Its connectorless electrodes directly protrude from a cuboid epoxy capsule containing all circuitry and power sources. This physical arrangement prevents fluid leaks or wire breakage and also simplifies and speeds implantation. Constant-current pulses of high compliance (34 volts) are delivered from a step-up voltage regulator under microprocessor control. A slowly pulsed magnetic field controls activation state and stimulation parameters. Program status is signaled to a remote reader by interval-modulated infrared pulses. Capsule size is limited by the two batteries. Silver oxide batteries rated at 8 mA-h were used routinely in 8 mm wide, 15 mm long and 4 mm high capsules. Devices of smaller contact area (5 by 12 mm) but taller (6 mm) were created for mice. Microstimulation of the rat's raphe nuclei with intermittent 5-min (50% duty cycle) trains of 30 μA, 1 ms pulses at 8 or 24 Hz frequency during 12 daylight hours lasted 21.1 days ±0.8 (mean ± standard error, Kaplan-Meir censored estimate, *n* = 128). Extended lifetimes (>6 weeks, no failures, *n* = 16) were achieved with larger batteries (44 mA-h) in longer (18 mm), taller (6 mm) capsules. The circuit and electrode design are versatile; simple modifications allowed durable constant-voltage stimulation of the rat's sciatic nerve through a cylindrical cathode from a subcutaneous pelvic capsule. Devices with these general features can address in small mammals many of the biological and technical questions arising neurosurgically with prolonged peripheral or deep brain stimulation.

## Introduction

Deep brain stimulation (DBS) is used in man to block the ongoing symptoms of various chronic neurological disorders, most commonly the tremor of Parkinson's disease and related forebrain disorders (Kringelbach et al., 2010; Oluigbo et al., 2012; Lozano and Lipsman, 2013). In a typical application, once the leads have been internalized successfully, stimulation is continuously applied for several years, which raises the possibility of inducing permanent changes. For example, activity-dependent axonal sprouting or synaptic potentiation may beneficially enhance the targeted pathway (Cooke and Bliss, 2006). Conversely, strong stimulation may produce harmful effects such as seizure foci (Albensi et al., 2007) or chemical, thermal and mechanical damage at the electrode-tissue interface (Cogan, 2008). Studying such effects over long periods in animal models is best done with implantable wireless stimulators. The main alternative is to tether the animal by electrically conducting cables to an external pulse generator, either continuously or for several hours daily, which risks breakage or entanglement of the intervening wires and distress to the subject.

Recent studies in rats have shown that prolonged stimulation of brainstem raphe nuclei can enhance recovery from traumatic brain or spinal cord injury, suggesting a treatment for early neurotrauma in man by interim DBS (Hentall and Burns, 2009; Hentall and Gonzalez, 2012; Carballosa Gonzalez et al., 2013). The experimental evidence was acquired with a robust stimulation system designed to deliver patterned electrical pulse trains to the brainstem of small mammals over many days. Full technical details on this device are presented here for the first time, including justification for critical choices entailed in the design. Some readily implemented modifications, such as for peripheral nerve stimulation in rats and stimulation of the mouse's brain, are briefly covered. Comparisons are drawn with other designs published in recent years, illustrating a range of solutions to the technical challenges presented by prolonged neural stimulation in small mammals.

## Methods

### Circuit

The schematic contains two integrated circuits (ICs): a 6-pin microprocessor (PIC10F206, SOT-23 package, Microchip Technology Inc.) and an 8-pin inductive step-up voltage regulator (LT3464, TSOT-23 package, Linear Technology Corporation). Both are of small footprint and possess the critical ability to enter a low-power “sleep” mode. Although these ICs have been available for about a decade, more recent devices offer little improvement for present purposes. Other important attributes of the 10F206 are an internal 4-MHz crystal clock, a low-power watchdog timer, the three output (I/O) pins and a dedicated input pin. Its memory is very small, consisting of 512 × 12 bits of program memory and 24 × 8 bits of data memory, but all functions that are critical to wireless stimulation can be programmed. Data memory was supplemented by reading or writing to unneeded control registers. Important attributes of the LT3464 are a dedicated feedback pin that allows easy configuration as a current-source, rapid recovery from sleep and an external analog input for optional setting of output amplitude. Its high output compliance (34 V) permits the use of high-impedance microelectrodes; thus a 1 megohm microelectrode can pass 34 μA, which stimulates the typical raphe neuron within a radius of approximately 0.2 mm, given a threshold-distance relation of 900 μA/mm^2^ (Hentall et al., 1984). This volume of excited tissue is sufficient for many experiments targeting specific nuclei in small mammals.

**Figure 1:**
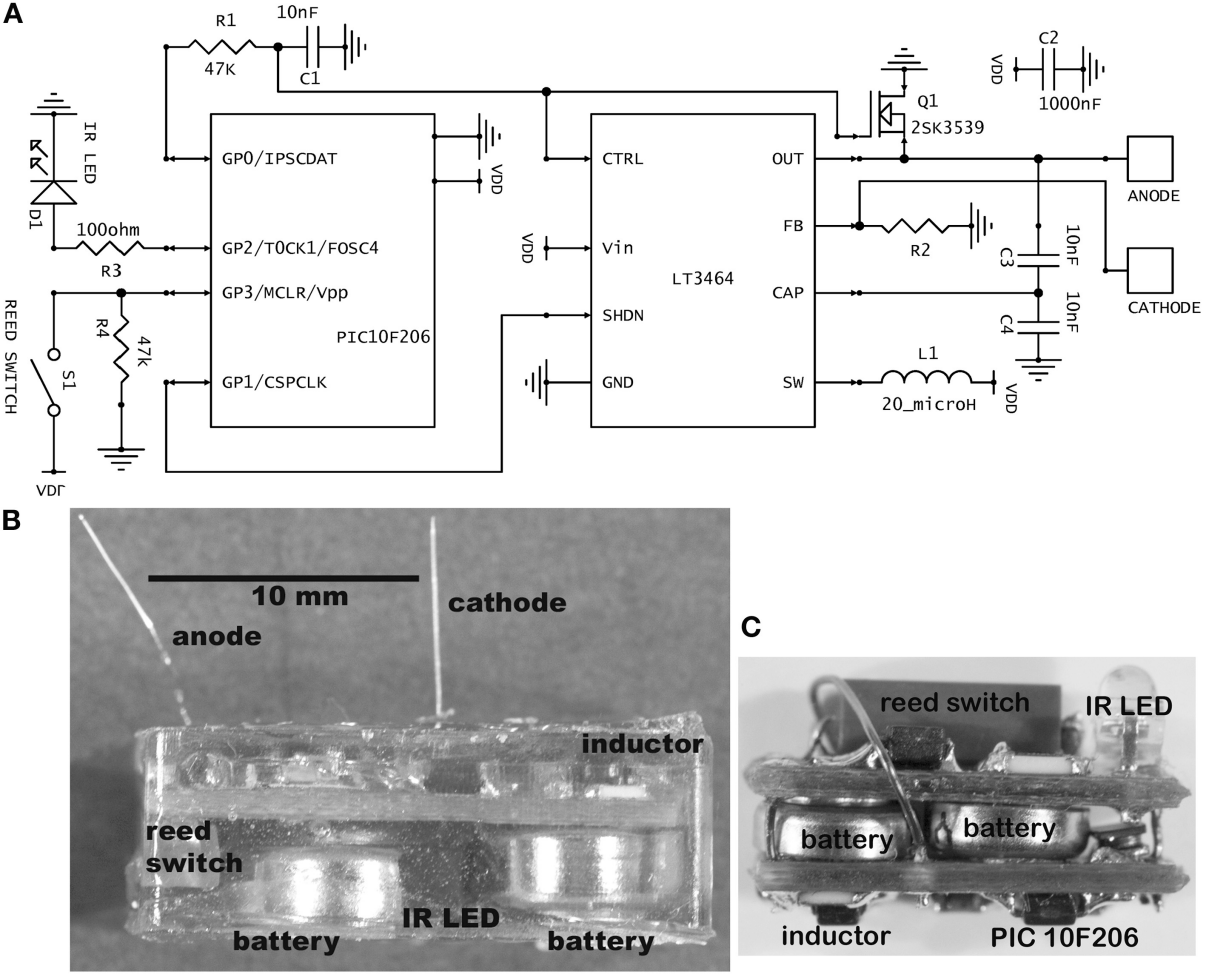
**(A)** Schematic of the stimulator circuit in the usual current-source configuration. The value for R2 was 42 kilohm for fixed amplitude stimulation (30 μA) and 10 kilohm for variable amplitude. In the alternative voltage-source configuration, R2 is 10 kilohm and an additional resistor of 40 kilohm is placed between the anode and cathode, giving a fixed 5-volt stimulus. **(B)** Photograph of a completed, epoxy-embedded stimulator containing the largest batteries used (type 392). Some partly visible components are labeled. **(C)** A mouse brain stimulator before embedding. This implementation contains the smallest batteries used (type 337). The photograph has the same scale as panel **(B)**.

**Figure 2:**
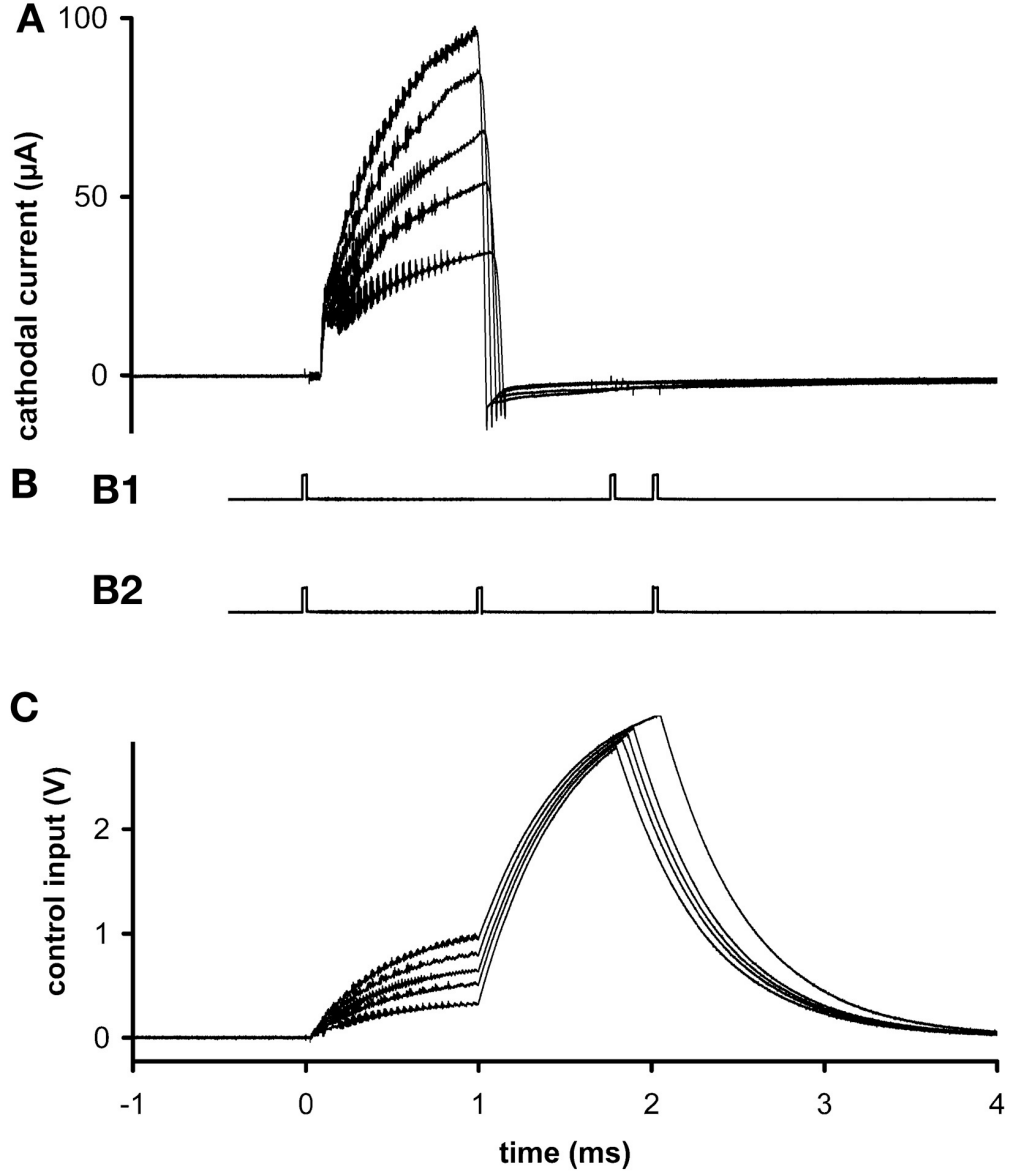
***Ex vivo testing***. **(A)** The stimulus current measured at 5 different stimulus amplitudes. **(B)** External signaling pulses sent during the highest amplitude current. The pattern alternated between **(B1)** and **(B2)** during the active stimulation phase. The first pulse interval in **(B1)** represents stimulus width plus clock time and the second represents stimulus amplitude; the first interval in **(B2)** represents pulse width and the second represents clock time plus amplitude. The alternation allows more than two parameters to be encoded with two intervals, to reduce power usage while transmitting at a reasonably fast update rate to the external reader. Inactive phases were signaled by the presence of four pulses, so there was no alternation of pattern. **(C)** Control voltages delivered during the stimulus pulses of graph **(A)**. All graphs have the same time base.

A magnetic reed switch (Coto Technology, CT05-1535-J1) serves as the transducer for remote control of stimulator settings. It passes no current when open and, given a suitably high value of in-series resistor, passes very low current when closed. Wireless output is signaled by an infrared (IR) light-emitting diode (Everlight Electronics Co. IR91-21C), using a customized pulse-interval code of 3 or 4 brief (20 μs) pulses. The current delivered by the LT3464 voltage regulator is set by a resistor (R2) attached to the FB pin (Figure 1A). The internal chip design provides the options of constant output when the voltage at the CTRL pin is >1.25 V or variable output when it is <1.25 V (Shtartgot, 2003). A roughly tenfold range of voltages is achievable for a fixed value of R2, from 34 V maximum to a minimum near the battery power supply. Most experiments using the fixed mode set the amplitude at 30 μA. Experiments that called for higher currents used the variable mode, with output programmed in 5 steps up to 100 μA (Figure 2C). The analog control voltage (0–1 V) is created by pulse-width modulation (PWM) of one of the microprocessor's output pins with an RC time-constant of 0.47 ms. Digital-to-analog (DAC) conversion by PWM minimizes the required size and number of components, compared with DAC or digital potentiometer ICs, for example. The CTRL pin also feeds the gate of an FET switch (Q1 in Figure 1A), which is closed by a higher voltage (>1.5 V), serving to truncate the otherwise slow output decay of the LT3464 step-up regulator after shutdown.

### Microprocessor programs and control

Programs are written in PICBASIC and placed in memory by MeLabs software using a U2 programmer board (all from microEngineering Labs Inc., Colorado Springs, CO). Magnetic input wakens the microprocessor from sleep mode to begin the stimulation pattern, which confers a long shelf life. After activation, the main program loops continuously, emitting one stimulus pulse per loop at a frequency of 8, 16, or 24 Hz. On every 16th stimulus pulse, 3 IR signaling pulses (20 μs) are emitted in the phase of active stimulation and 4 pulses in the rest phase of the duty cycle (Figure 2B). During most of this loop, the microprocessor and voltage regulator are in sleep mode. The watchdog timer's periodicity determines the stimulation frequency and clock time.

Magnetic input detected during the main cycle initiates a subroutine that looks for additional slow magnetic pulses for the next several seconds. Depending on the number of pulses received, various stimulus parameters are changed, the clock is synchronized to real time or the device is returned to the inactivated state. The stimulation pattern in most experiments was a 5-min 50% duty cycle (alternating rest and stimulation) applied daily during 12 daylight hours. The rationale for this pattern was to avoid transmitter depletion with the 5-min rest periods and to prevent disruption of diurnal arousal cycles with the nighttime rest. The main program loop continues to signal and keep time during these short and long rests.

A handheld external control box detects and decodes the infrared pulses emitted by the stimulator. Pulse width, clock time, stimulus frequency, pulse amplitude and program status (stimulating or resting) are written to a liquid crystal display. The control box can optionally feed an electromagnet probe with programmed pulses to change the stimulator settings, although normally a handheld bar magnet is more easily deployed to create pulses. The nearby presence (<2 cm) of the magnetic field stops the program immediately; the number of times that the field disappears and returns within a 2 s period represents the code. In a typical software realization, one pulse resets the clock to midday (used for synchronizing the internal clock to real time), two pulses cycle through 5 available stimulus amplitudes or pulse widths, three pulses alternate between frequencies, 4 pulses add 1 h to the clock and more than 4 pulses inactivate the device.

### Physical construction, microelectrodes, and power source

Components are soldered onto standard, two-sided, 0.031-thick FR4 printed circuit board (PCB) fabricated commercially. Resistors and capacitors are all surface-mount (size 0603). The circuit is powered by two 1.55 V silver oxide coin cells, joined in series by a metal strip. They are attached by silver-based conductive epoxy (8331-14G, MG Chemicals, Surrey, BC, Canada). Both the cathodal microelectrode and the stainless steel wire anode are soldered into through-holes on the PCB. The microelectrodes are cut to a fixed length so that they protrude from the epoxy capsule to reach the targeted brain region with minimal clearance above the skull. The microelectrodes used were made from either platinum–iridium (PI20030.5A5: Microprobe, Inc., Gaithersberg, MD) or tungsten (#573210: A-M Systems, Sequim, WA). Both types were insulated with Parylene C, rated by the manufacturers to have an “AC impedance” of 0.5 megohm and roughly 0.13 mm in shaft diameter. Platinum–iridium is biologically and chemically less reactive than tungsten, and the platinum–iridium microelectrodes never produced histological signs of damage after 8 weeks in the rat's midbrain (Carballosa Gonzalez et al., 2013). The tungsten microelectrodes are less expensive and their greater rigidity provides better stereotaxic accuracy in targeting deeper parts of the brain.

For encapsulation, a low-conductance biocompatible epoxy mixture (DP-270, 3 M Electronic Specialty, St. Paul, MN) is allowed to harden for 24 h or more in a suitable flexible mold. Batteries rated at 8 mA-h (at 20°C, under specific constant loading) were most often used (Renata or Energizer 337; diameter 4.8 mm, height 1.6 mm). For routine implantation in rats, these were packaged in a capsule 8 mm wide, 15 mm long, and 4 mm high, which weighed 1.2 ± 0.1 gm. To achieve longer lifetimes, devices with 44 mA-h batteries (Energizer 392) were fabricated (Figure 1B), necessitating longer (18 mm) and taller capsules (6 mm). For mice, a smaller cranial contact area was called for, so a two-board sandwiched design of 5 mm width, 12 mm length and 6 mm height was created, containing 8 mA-h batteries (Figure 1C).

An energy budget can be calculated from manufacturer's specifications. Assuming battery power of 3 V, the microprocessor draws 240 μA when operating and 1 μA in the sleep state with an active watchdog. The step-up regulator draws 25 μA when operating open-circuited and 0.5 μA in the sleep state. The IR LED draws 20 mA per 20 μs pulse. From these figures, trains of 1-ms, 30-μA stimulus pulses given at 8 Hz for 12 h daily with a 50% duty cycle cause an average daily current flow of 12 μA, implying a 56-day lifetime with two 8 mA-h batteries.

### Animal experiments

All work on animals followed protocols approved by the Institutional Animal Care and Use Committee of the University of Miami, Miller School of Medicine. As described more fully elsewhere (Hentall and Gonzalez, 2012; Carballosa Gonzalez et al., 2013), stimulators were implanted in male or female adult Sprague-Daley rats (250–320 g) under isoflurane anesthesia (1.2%) delivered by face mask, using stereotaxic guidance. The capsules were fixed by dental cement to 3 small stainless steel screws (00–90, 1/16th inch, Antrim Miniature Specialties, Fallbrook, CA) in the skull. The anode wire was wrapped around one of the screws and tied there with suture thread before the cementing. The cranial incision was left open to assist wound healing and avoid excessive stretching of face skin around the implant.

For peripheral nerve stimulation, the same method of anesthesia was used for implantation and electrophysiological testing. Hind leg incisions were closed completely over the capsule by suturing; the IR pulses passed through approximately 0.5 cm of tissue and were readily detectable. The cathode was a 16-gauge ring electrode of 5 mm length that was insulated on the outside by heat-shrink tubing, fed by an insulated stainless steel wire. This electrode was slipped over the cut nerve. A metal eye protruded from the epoxy-embedded capsule to allow subdermal attachment by suturing in the hip region. The anode, a bare stainless steel wire, was positioned subcutaneously.

## Results

### Working lifetime of devices *in vivo*

The lifetime of activated stimulators (*n* = 128) with 337-type (8 mA-h) silver oxide batteries was analyzed from three series of experiments. All used trains of pulses (1 ms, 30 μA) that were alternated with 5-min rests for 12 h daily. In one series (*n* = 58), stimulation was applied for 7 days through platinum–iridium microelectrodes to the midbrain's dorsal raphe or median raphe (Carballosa Gonzalez et al., 2013). The frequency was 8 Hz (*n* = 49) or 24 Hz (*n* = 9). Stimulation began 4 h (*n* = 49) or 1 week after device implantation. The second series (*n* = 38) tested different durations of stimulation (usually 7 or 14 days) applied through tungsten microelectrodes in the hindbrain's nucleus raphe magnus, starting 0 or 2 or 7 days after implantation (Hentall and Gonzalez, 2012). In the third series (not yet published), 1 week (*n* = 20) or 3 weeks (*n* = 12) of stimulation was delivered through the tungsten microelectrodes to the midbrain periaqueductal gray matter, beginning 0, 2, or 21 days after implantation. Electrical failures, defined as unplanned permanent cessation of the infrared signaling pulses, occurred in 21 of the 128 devices. Kaplan-Meir estimation using this censored data gave a mean lifetime of 21.13 days (s.e.m. 0.79; estimated with SPSS version 21, IBM Corp.). Although this lifetime is less than predicted by the battery specifications, high current transients and temperatures nearer to body temperature are some of the factors that may explain the difference. There was no significant dependence on activation delays or different frequencies; indeed stimulators operating at the higher frequency (24 Hz) never failed during 7 days of operation. Mechanical or chemical failure of the transparent epoxy capsule or of its visible internal contents was never observed.

To achieve longer lifetimes, placement of batteries outside the cranially attached capsule was first explored. Lithium batteries, rated at 120 mA-h (BR-1632A; Panasonic-BSG) were implanted subcutaneously and connected by leads to the cranially attached stimulator in some rats (*n* = 6). The leads were made of insulated MP35N, a strong and ductile cobalt-nickel-chromium alloy with added molybdenum that has often been used for commercial pacemaker leads (Ratner et al., 1996). However, frequent breakage or disconnection of leads at their insertion points, perhaps made more likely by the rat's high flexibility and motility, compelled us to discard this approach. The desired outcome was more readily achieved by unitary encapsulation of circuitry and batteries as in the original design. Using this approach, a fourth series of studies examined chronic PAG stimulation in rats with spinal cord injury. Active cranial stimulators with the 44 mA-h batteries in the larger capsules (*n* = 16) passed 70 μA pulses with the standard intermittent pattern for 6 weeks without electrical or internal mechanical failure.

### Output characteristics

In *ex vivo* tests, different stimulus amplitudes were applied to microelectrodes in phosphate-buffered saline at 20°C. Current was measured at resistor R2. Flicker can be seen superimposed on the output (Figure 2A), due to the rapid switching of the inductor and the creation of control voltages by PWM (Figure 2C). These oscillations do not significantly hinder neuronal excitation, since the typical membrane time-constant is about 1 ms, and they can be removed if desired by filtering, at the expense of slightly longer rise times. Current density was never found to be a limiting factor, neither in platinum–iridium nor in tungsten microelectrodes, when stimulus amplitude ranged up to 100 μA (at 8 Hz) and frequency ranged up to 24 Hz (at 30 μA) (e.g., Figure 2A). From the area of the exposed conical tips of tungsten microelectrodes seen under a microscope, charge density was estimated to be 0.5 mC/cm^2^.

### Adaptation for constant-voltage peripheral nerve stimulation

The basic circuit can readily be adapted to other uses. For example, a voltage source is preferable with gross electrodes, because applied tissue potentials can be estimated and voltage is one mathematical step closer to field strength, the operative parameter in extracellular far field stimulation (Coburn, 1989). A voltage source is obtained simply by placing a resistor in parallel with the anode and cathode; for example, a 40 kilohm resistor with a 10 kilohm resistor at R2 allows a 5 V stimulus.

**Figure 3:**
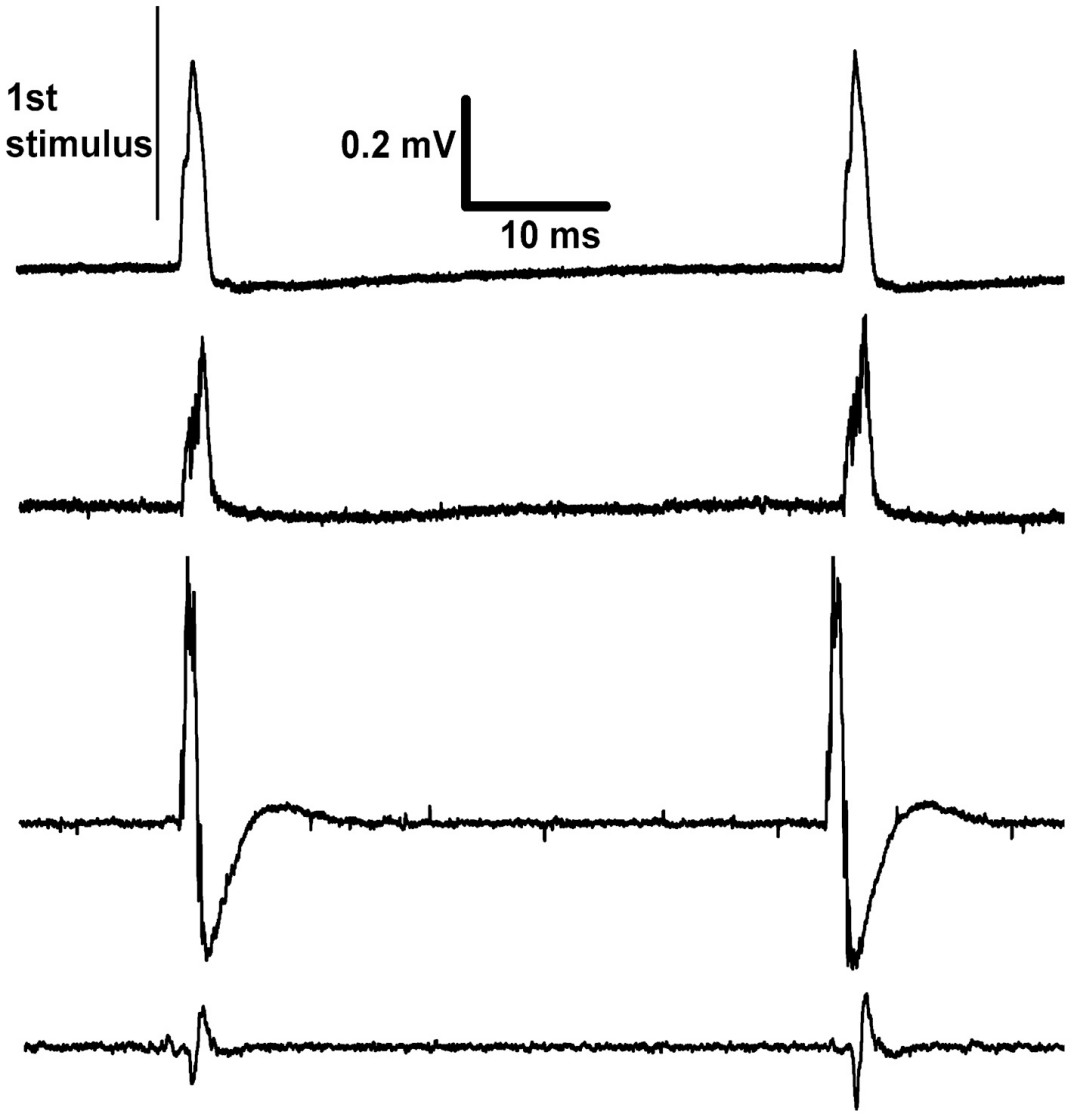
**Recordings made differentially from dorsal rootlets of lumbar segment L4**. The proximal end of the cut sciatic nerve was stimulated at approximately 20 Hz. Each trace is from a different rat recorded on the last day of continuous stimulation and shows two responses per trace. The differences between traces in the sizes and shapes of evoked response are explained by their dependence on factors such as the positioning of the paired recording electrodes and inter-electrode conductance.

This voltage-source circuit was used to stimulate the sciatic nerve in rats. Pulses of fixed amplitude and width (5 V, 190 μs) were given for three days (*n* = 2) or one day (*n* = 2) at 20 Hz. At the end of experiments, electrical recordings were made on L4 dorsal rootlets with hook electrodes. The results showed that the stimulus parameters were sufficient to activate Aβ fibers but not Aδ or C fibers (Figure 3). These recordings confirmed the continued functional viability of the devices. The viability of the brain stimulators had been previously shown with the histological marker 2-deoxyglucose (Carballosa Gonzalez et al., 2013). No evoked twitches were observed, but the denervated foot assumed a more elevated position during stimulation. These implants were physically secure in the rats for all days of observation.

## Discussion

The range of design choices for implantable small-animal stimulators has been fairly stable over the last decade, given the state of the art in electronics, batteries and electrodes. Hence, published designs and specifications can be reasonably compared. Non-commercial designs are typically optimized for quite specific experimental applications, but sometimes can be used more generally, as is the intention with commercial devices. A number of designs for brain stimulation have been reported with detailed specifications (Table 1). Similar devices have been described in less detail for long-term peripheral nerve stimulation (Al-Majed et al., 2000; Lee et al., 2010).

**Table 1:** **Comparison of some recent brain stimulators for small animals**.

**PARAMETER**	**DEVICES**				
Information source	Present article	*de Haas et al., 2012*	*Arfin et al., 2009*	*Millard and Shepherd, 2007*	*Harnack et al., 2008*
Species studied:	Rat	Mouse	Zebra finch	Rat, mouse	Rat
Typical active lifetime	21 days or >42 days	10 h	12 days	Indefinite	21–35 days
Battery	Ag-Ag_2_O (2)	Ag-Ag_2_O (3)	Li (2): ML621+614	None (inductive)	Ag-Ag_2_O (2)
Tunneled lead to head	No	Yes	No	Yes	Yes
Output range	20–100 μA	20–100 μA	10–1000 μA	100–500 μA	50–600 μA
Compliance	34 V	4.65 V	5 V	5 V	12 V
Independent channels	1	2	4	1	2
Frequency	8, 16, or 24 Hz	131 Hz	Large range	50–5000 Hz	131 Hz
Pulse width	100–1000 μs	60 μs, fixed	180 μs/phase, fixed	25–250 μs/phase	52 μs, fixed
Biphasic	No	Yes	Yes	Yes	Yes
Control medium	Magnet	Magnet	Electromagnetic	Induction coil	Electromagnetic
Functions controlled	Various	On/off	Various	Various	Various
Output medium	Infrared	Infrared	Visible LED	Visible LED	Electromagnetic
Status readout	Program variables	On/off, saturated	On/off	On/off	Program variables
Length, width, height (in mm) of main device	15, 8, 4 or 18, 8, 7	30 × 8 (diameter)	13, 13, 17^*^	14, 12, 16 (mouse version)	38, 20, 13
Weight (g)	2.0	2.1	1.3	2.5 (mouse version)	13

* Estimated from photographic scale.

A major consideration for the present design was maximization of lifetime for chronic stimulation. This implies both physical durability and a long-lasting power source. Durability in the present device was achieved by one-piece encapsulation of the batteries, circuitry and electrode attachments within high-strength, high-resistivity epoxy. The physical integration of the stimulator and electrodes also speeds and simplifies the surgery; the stimulator is placed on the skull and the electrode stereotaxically positioned in the brain in one maneuver. Capsule size should be minimized for the animal's comfort and because overhang or excessive material above the head risks physical detachment. The non-reusability of the encapsulated assembly containing non-rechargeable batteries was a disadvantage, but its assembly is very rapid and all components are inexpensive, electrodes being the most expensive item (unless made in the laboratory).

A common alternative for attaining longer battery lifetimes involves locating some or all of the circuit components, including batteries or inductive receiver coils, distant from the skull, such as subcutaneously in the back, shoulder, or abdomen, (Millard and Shepherd, 2007; Harnack et al., 2008; Arfin et al., 2009; de Haas et al., 2012). As in commercial DBS devices, subcutaneously tunneled wires connect the electrodes with the remote components. However, tunneled leads, even when coiled, are subject to repeated bending that increases the potential for breakage and for electrolyte penetration at connections (causing corrosion or short circuits). In initial explorations for this project, breakage was frequent at attachment points. Also, the wires were quickly surrounded by new connective tissue that was quite hard and thus restricted their flexing. If leads do not break, they are presumably sometimes uncomfortable for the animal. Lead breakage near fixed implants is reported to be a common cause of failure in rigorously engineered DBS systems for humans (Alex Mohit et al., 2004; Blomstedt and Hariz, 2005), who can at least reduce the risk by voluntarily limiting movement.

Another approach to power drain is to affix temporarily a more obtrusive device containing larger batteries on the animal's head. A means of rapid connection is then needed between the temporary part (batteries with or without electronics) and the cranial implant (electrodes with or without electronics). Another way to extend battery lifetime is by arranging for inductive recharging, done either *in situ* or after temporarily detaching the device. This method comes at the cost of somewhat greater electronic complexity and size. Alternatively, batteries can be omitted and power be provided by induction alone (Millard and Shepherd, 2007), which offers complete flexibility of stimulation pattern, since this is set by the circuits driving the induction coils. A limitation is that the external coils must be near the subject.

Remote two-way communication is very useful in wireless stimulators. In their simplest form, input signals can turn the device on and off, which can save power or remove adverse effects (de Haas et al., 2012). Output signals may simply indicate on or off status (Millard and Shepherd, 2007; de Haas et al., 2012). More elaborate controls can use instantaneously transmitted commands (Millard and Shepherd, 2007; Harnack et al., 2008) or set-and-go methods, in which parameters must be modified by temporarily halting the experiment (Arfin et al., 2009; de Haas et al., 2012). In the present design, pulse width, amplitude, and frequency are all monitored continuously and may be altered with a few taps of a nearby magnet during a brief period of intervention. An additional feature is the ability to reset the internal clock, which can be used to counter drift in the RC watchdog timer. This is particularly useful in studies of arousal-related areas, which need a fixed diurnal stimulation cycle. In other designs, reprogramming is done by temporarily connecting the device via cable to a computer, as in a commercial device that is detached temporarily for inductive recharging (2 Channel Wireless Stimulator, v 1.1; Triangle BioSystems, Inc., Durham, NC, USA).

Remote communication has a significant impact on power drain, which depends on the medium (e.g., RF, IR, low-frequency magnetic field) and the desired range. Far-field RF consumes a lot of power but offers long-distance multidirectional communication that is less affected by physical barriers. The pulsed directional IR beam of the presently described stimulator was designed for minimal power drain, but it has a short range and is highly directional. Power consumption is also affected by the choice of stimulation parameters. Charge delivery should be set as low as possible for the specific application. In the present device, longevity was enhanced by the use of intermittent stimulation, although other considerations influenced this choice. Thus a fairly low stimulus frequency (8 Hz) was used to match the regions' slowly firing neurons; interference with arousal was reduced by a 12-h nocturnal pause; depletion of synaptic release or firing fatigue was handled by imposing 5-min gaps in a 50% duty cycle between trains.

Other critical trade-offs involve the number of electrodes and independent channels, which influence size, power usage and physical and electrical complexity. The present device is minimal in this respect, passing monophasic pulses through a centrally located monopolar cathode and a return wire anode. Multiple electrodes have various experimental advantages, such as alternative targeting or synchronous stimulation of bilateral structures, although in many experiments they are superfluous. A one-channel design can be adapted to multiple cathodes by feeding them in parallel, but this has the drawback that the relative stimulus current is inconveniently fixed by the ratio of electrode conductances. Some devices use biphasic pulses and some in addition offer independent control of width and amplitude for each phase (Harnack et al., 2008), which may be useful for reducing artifact in electrical recording. However, the opposite phases do little to reverse chemical deposition or other electrochemical reactions in metal electrodes, which are very far from equilibrium (Cogan, 2008). Additionally, closely spaced bipolar electrodes can be used to limit the region of stimulation. Close spacing is essential for gross electrodes, which create an approximately constant field that will cause massive non-local stimulation if, for example, separation is several centimeters (not uncommon). The radial fields created by punctate monopolar microstimulation accomplish this localization with less invasiveness and without introducing the complexities of anodal block (Hentall, 1987, 1989). However, the higher impedances of microelectrodes demand a higher output compliance; the 34 V compliance of the present device is the highest in Table 1.

Much ingenuity continues to be given to the replacement of traditional electrical brain stimulation with alternative methods of activation that are less invasive or more selective for cell phenotype. Among interesting non-invasive solutions, direct stimulation by ultrasound or by infrared light can target deep regions (Bystritsky et al., 2011; Richter et al., 2011), but their transduction is of uncertain causation and possibly harmful to cells or tissue. External magnetic stimulation has frequently proved useful, although it cannot precisely address discrete brain areas, especially the deeper areas (Hallett, 2000). Insertion into target tissue of sub-millimeter transducers that convert light or magnetic fields into electrical fields is another intriguing possibility (Abdo et al., 2011; Kane et al., 2011; Bonmassar et al., 2012; Kim et al., 2013). Many of the recent novel approaches involve insertion of genes expressing membrane receptors responsive to light or to chemicals that are not normally present (Miesenbock and Kevrekidis, 2005). Nevertheless, the long-term provision of stimulation through wearable devices to unattended wild-type animals, free to move within a reasonably comfortable space and unrestricted by proximity to a bulky apparatus, is still best done by passing current directly into inserted electrodes. The limitations of space and proximity imposed by battery size or power induction will disappear if methods to tap biological energy by electrical conversion become practical for long-term use (Heller, 2006; Romero et al., 2009). Refinement of this methodology, exploiting advances in the miniaturization and biocompatibility of power sources, electronics and electrodes, thus remains a productive approach.

### Conflict of interest statement

The author declares that the research was conducted in the absence of any commercial or financial relationships that could be construed as a potential conflict of interest.
